# Hypersensitivity reaction to rivaroxaban with a successful switch to apixaban: A case report

**DOI:** 10.1002/ccr3.9213

**Published:** 2024-07-23

**Authors:** Keyhan Mohammadi, Ramin Ansari, Shakila Yaribash

**Affiliations:** ^1^ Department of Clinical Pharmacy, Faculty of Pharmacy Tehran University of Medical Sciences Tehran Iran; ^2^ Research Center for Antibiotic Stewardship and Antimicrobial Resistance, Imam Khomeini Hospital Complex Tehran University of Medical Sciences Tehran Iran; ^3^ Faculty of Pharmacy Tehran University of Medical Sciences Tehran Iran

**Keywords:** apixaban, hypersensitivity, rash, rivaroxaban

## Abstract

**Key Clinical Message:**

The precise management of hypersensitivity reactions to direct oral anticoagulants (DOACs) and the potential for cross‐reactivity among different DOACs remain unclear. In such cases, switching between DOACs may be feasible and could be considered, but close monitoring for adverse effects is essential, tailored to individual patient responses and tolerability.

**Abstract:**

Hypersensitivity reactions to DOACs, though considered rare, have been documented. This report describes the case of a 28‐year‐old male with a history of testicular cancer who was recently diagnosed with deep vein thrombosis. He was referred to an outpatient pharmacotherapy clinic due to suspected rivaroxaban‐induced cutaneous reactions. Following a thorough evaluation, his anticoagulant therapy was switched from rivaroxaban to apixaban. This change was successfully implemented, and no hypersensitivity symptoms recurred during subsequent follow‐up. This case demonstrates the importance of recognizing potential adverse reactions to DOACs and illustrates the feasibility of switching anticoagulants under close medical supervision to ensure patient safety and effective treatment.

## 1 INTRODUCTION

The role of direct oral anticoagulants (DOACs) such as apixaban and rivaroxaban in the management of cancer‐associated thrombosis (CAT) has suggested promising outcomes compared to previous anticoagulants, including low‐molecular‐weight heparin. DOACs are at least as effective as heparin derivatives and are preferred as an oral agent in managing cancer‐associated venous thromboembolism (VTE).^1^ While DOACs represent a significant advancement in anticoagulation therapy for cancer‐associated VTE, challenges have been reported during DOAC treatment, specifically in terms of hypersensitivity reactions.^2^ These reactions, though rare, are documented in studies^3^ and most commonly present as erythematous and pruritic eruptions, urticaria, and angioedema.^4^ However, the incidence of hypersensitivity reactions in the literature is not properly defined.^5^

This case report highlights practical challenges and considerations in managing a possible hypersensitivity reaction to DOACs in cancer‐associated VTE. The case illustrates the complexities of selecting appropriate DOACs, particularly regarding potential cross‐reactivity.

## 2 CASE HISTORY AND EXAMINATION

A 28‐year‐old male with a history of testicular cancer was prescribed 5 mg of apixaban, administered twice daily, for a recent VTE episode. Ten days after initiating apixaban, 10 mg twice daily for 7 days, followed by 5 mg twice daily, the patient consulted his oncologist regarding experiencing dermatological issues. The patient displayed symptoms of mild and brief itching without any rash, which improved naturally during ongoing apixaban therapy, although it was likely not related to the medication. Nonetheless, he was referred to a cardiologist for further evaluation.

Based on cardiologist consultation, the apixaban was discontinued and the rivaroxaban 20 mg daily was initiated at the time of the next scheduled dose of the apixaban, 12 h after the last dose of apixaban. Following rivaroxaban treatment after 5 days, the patient complained of dermatologic presentations characterized by maculopapular rash and generalized itching. The lesions were noted to disappear under pressure. Given the patient's history and the physician's assessment, rivaroxaban‐induced hypersensitivity was diagnosed.

## 3 METHODS

Accordingly, after pharmacotherapy consultation, rivaroxaban was discontinued and replaced with apixaban at the next scheduled dose. The patient was referred to the pharmacotherapy clinic for further management of anticoagulation therapy. The Naranjo Adverse Drug Reaction Probability Scale score^6^ for rivaroxaban was 5, classifying the reaction as “probable.”

## 4 CONCLUSION AND RESULTS

Two days later, the patient's symptoms gradually subsided and were mostly resolved by the time of his visit to the pharmacotherapy outpatient clinic. Only very mild itching remained. Laboratory data are provided in Table 1. Symptomatic treatment was provided for a few days, including oral fexofenadine 180 mg per day, topical calamine, topical zinc oxide, and a topical moisturizer applied twice daily to the affected area. Apixaban therapy was continued.

**TABLE 1:** The laboratory data of the reported case.

Test	Result	Unit	Reference range
WBC	7.2	10^3^ μL	4–11
RBC	4.16	10^6^ μL	4.3–5.6
HGB	13.3	g/dL	14–17
HCT	38.8	%	39–53
MCV	88.5	fL	80–96
MCH	29.6	pg	27–33
MCHC	33.4	g/dL	30–38
Platelets	244	10^3^ μL	150–450
RDW‐CV	12.7	%	11–15
Urea	25	mg/dL	19–44
Creatinine	1.0	mg/dL	0.7–1.4
Ferritin	142	ng/mL	Males: 16–220
TSH	1.4	μIU/mL	0.4–5
E.S.R 1 h	11	mm/h	Up to 15

The patient's symptoms completely resolved within a few days and did not recur during follow‐up visits in the subsequent weeks.

## 5 DISCUSSION

Since the introduction of DOACs in the early 2000s, various adverse effects have been reported, including hypersensitivity reactions. These reactions can range in severity and presentation. It is important to recognize that drug‐induced skin reactions are relatively rare in the general population and are often a diagnosis of exclusion. However, they can account for up to one‐third of all medication‐related adverse reactions.^7^

The ROCKET‐AF study supports findings that rivaroxaban has a hypersensitivity reaction rate of 0.1%–1%, classified as an uncommon adverse reaction. There are reports of truncal maculopapular exanthema associated with neutrophilia and mild eosinophilia, toxic skin eruption, cutaneous/leukocytoclastic vasculitis, erythema multiforme, rash, pruritus, skin necrosis, angioedema, and anaphylaxis or anaphylactic shock with rivaroxaban.^8^ According to the eHealth database of 11,904 people who reported side effects to apixaban, 160 patients (1.34%) reported experiencing rash between 2015 and 2020.^9^ The ARISTOTLE study found that hypersensitivity reactions occur in less than 1% of patients taking apixaban.^10^

Following a hypersensitivity reaction to a DOAC, recommendations include discontinuing the DOAC, providing supportive care, and, if possible, switching to a different anticoagulant. However, the indication for anticoagulation should be reevaluated in each case. Some reports have shown that discontinuing the DOAC without substitution (e.g., in cases of rivaroxaban‐induced leukocytoclastic vasculitis) has not resulted in negative clinical outcomes.^11^ There are reports regarding switching from apixaban to warfarin in a case of cutaneous leukocytoclastic vasculitis,^12^ anaphylactic reaction,^10^ and acute generalized exanthematous pustulosis.^13^ Moreover, several reports exist regarding the successful switch from rivaroxaban to low molecular weight heparin (LMWH) in the case of generalized rash^14^ maculopapular erythematous and itchy rash,^15^ and anaphylactic reaction.^16^

Limited information is available regarding the mechanisms and immunological aspects of DOAC‐induced hypersensitivity reactions and cross‐reactivity between agents. Some theories suggest that rivaroxaban's small molecular size may not trigger an immune response, and its distinct structure from apixaban may prevent cross‐reactivity.^17^ Since hypersensitivity reactions may not occur with all DOACs, switching to an alternative agent, such as apixaban, could be considered to evaluate clinical outcomes. However, limited research exists on switching between DOACs in cases of hypersensitivity reactions. Table 2 summarizes data from the literature review on previous reports of DOAC‐induced hypersensitivity reactions and cross‐reactivity.

**TABLE 2:** Previous reports regarding hypersensitivity and cross‐reactivity between apixaban, rivaroxaban, and dabigatran.

No.	Case	Indication	Probable/definite causal agent, dose	Time from the first dose	Clinical manifestations	Decision on anticoagulant	Outcome (s)	Ref.
1	63‐year‐old man	Pulmonary embolism	Rivaroxaban, 15 mg twice daily for 21 days	10 days	Urticaria, hand edema, and itching, and angioedema	Dabigatran 150 mg twice daily	The patient developed pruritic morbilliform exanthema eruptions 10 days later	^17^
2	61‐year‐old woman	Orthopedic surgery	Rivaroxaban, 10 mg daily	10–15 min	Anaphylactic reaction	Dabigatran 150 mg twice daily	The results of provocation tests confirmed the safety of dabigatran	^18^
3	52‐year‐old woman	Pulmonary embolism	Apixaban, 10 mg twice daily	2 days	Purpura and swelling in the lower limbs (Diagnosed as cutaneous leukocytoclastic vasculitis)	Dabigatran 150 mg twice daily	No flare‐up of purpura	^19^
4	62‐year‐old man	Protein C deficiency	Apixaban, 5 mg twice daily	6 h	Hemorrhagic pruritic rash	Rivaroxaban 10 mg daily	The Rash continued to worsen. The patient was stabilized on Warfarin	^2^
5	45‐year‐old male	Deep vein thrombosis/ pulmonary embolism	Rivaroxaban, 15 mg twice daily for 21 days	7 days	Petechial rash and palpable purpura.	Apixaban 5 mg twice daily	Similar reaction with Apixaban. The Patient was finally stabilized on warfarin	^20^
6	48‐year‐old man	Post‐cardiac stent implementation	Apixaban (Dose unavailable)	5 days	Palpable purpura on his lower legs, diagnosed as IgA vasculitis.	Rivaroxaban (Dose unavailable)	No further lesion development while on rivaroxaban	^10^
7	60‐year‐old female	Atrial fibrillation	Apixaban, 5 mg twice daily	11 days	Non‐vertiginous dizziness without syncope, severe pressure‐type headache, diplopia, confusion	Rivaroxaban 15 mg twice daily	No similar or other adverse symptoms 4 months after rivaroxaban initiation	^21^
8	60‐year‐old man	Atrial fibrillation	Apixaban, 5 mg twice daily	10 days	Burning, pruritic, petechial rash confirmed as leukocytoclastic small‐vessel vasculitis	Rivaroxaban 15 mg twice daily	During the three‐week visit, the patient remained asymptomatic	^22^
9	79‐year‐old female	Atrial fibrillation	Rivaroxaban 20 mg daily	Several days (no exact data available)	Rash and pruritis	Apixaban (dose unavailable)	No further adverse reaction was experienced while on apixaban	^23^

A case involving a 63‐year‐old man reported cross‐reactivity between DOACs. After starting rivaroxaban, the patient experienced urticaria, hand edema, and itching, which progressed to angioedema, leading to the discontinuation of the drug and dabigatran initiated, but the same symptoms, along with a morbilliform exanthema, reappeared within 10 days. Symptoms resolved a week after stopping dabigatran, and the patient successfully switched to warfarin with enoxaparin without further incidents.^18^ In contrast, a 61‐year‐old woman experienced an anaphylactic reaction to rivaroxaban. Notably, she was successfully switched to dabigatran without any further adverse reactions.^19^ This case suggests that cross‐reactivity between rivaroxaban and dabigatran may not always occur, and in some cases, an alternative DOAC can be adminitered safely.

A report describes apixaban‐induced purpura and swelling in the lower limbs of a 52‐year‐old woman after 2 days of initiating apixaban. This presentation was suggestive of cutaneous leukocytoclastic vasculitis (LCV), although this was not confirmed by biopsy.^24^ Enoxaparin was substituted for the drug, and prednisolone was started. The purpura diminished after 24 days with no recurrence of any reactions.

Moreover, Cortellini et al. suggested a possible cross‐reactivity between edoxaban and apixaban.^20^ Conversely, reports suggest cross‐reactivity between apixaban and rivaroxaban. Notably, a 62‐year‐old man with protein C deficiency, previously on warfarin, developed a hemorrhagic pruritic rash around his buttocks and groin 6 h after switching from warfarin to apixaban. When switched to rivaroxaban the next day, the rash worsened. Symptoms resolved within 24 h after returning to warfarin. In this case, rivaroxaban was initiated before apixaban was fully cleared from his system, making the causal relationship between rivaroxaban and the worsening symptoms unclear.^2^

In a case involving a 45‐year‐old male, a petechial rash and palpable purpura developed on his upper and lower extremities 1 week after switching from rivaroxaban to apixaban. After several months on rivaroxaban, the cause of the skin reaction remained uncertain. Both rivaroxaban and apixaban were discontinued, and the patient was switched to warfarin without any further issues. A biopsy later confirmed seronegative LCV.^25^

In another report, a 74‐year‐old woman developed a progressive rash on both lower extremities 23 days after starting apixaban for VTE. She was switched to rivaroxaban without any recurrence and was eventually diagnosed with LCV.^21^

Similar results were seen in a case of apixaban‐induced lichenoid eruption in a 78‐year‐old male with atrial fibrillation (AF) who developed a rash on both upper extremities. Due to submandibular gland excision, apixaban was held for 5 days, and the rash resolved but reappeared after apixaban was restarted. The patient was switched to rivaroxaban and denies any further rash or itching symptoms.^9^

Similarly, a 48‐year‐old man developed palpable purpura on his lower legs five days after starting apixaban. After switching to rivaroxaban, he had no further lesions, and a biopsy confirmed IgA vasculitis. In another case of IgA vasculitis, a patient with a history of hypersensitivity to rivaroxaban developed a rash on their arms, legs, and abdomen 2–3 weeks after starting apixaban. The patient's condition eventually stabilized on warfarin.^10^ Other reports describe successful switches from apixaban to rivaroxaban.^22^, ^23^

In a report, a 79‐year‐old female with AF developed rash and pruritis in her breasts, upper extremities, and trunk several days after rivaroxaban initiation. Despite taking diphenhydramine, her symptoms did not improve until rivaroxaban was stopped, and then successfully switched to apixaban without any further reaction.^26^

While the specific brand of drug used in this case is unknown, hypersensitivity to a formulation's excipient is a potential explanation. Polyethylene glycol (macrogol), found in all strengths of Xarelto tablets, has been linked to hypersensitivity reactions, as reported in a 61‐year‐old man who developed a rash and itching while taking the medication for VTE prophylaxis.^27^

Given the rising global use of DOACs and the potential for hypersensitivity reactions as an adverse effect, further research is needed to identify the optimal alternative agents in such cases and assess the likelihood of cross‐reactivity between DOACs. While our case featured mild itching during apixaban treatment, the likelihood of apixaban causing this reaction seems low, as the patient tolerated it well upon reintroduction. Using the Naranjo adverse drug reaction probability scale, rivaroxaban scored 5, indicating a possible causal relationship with the observed clinical events.^6^

**FIGURE 1:**
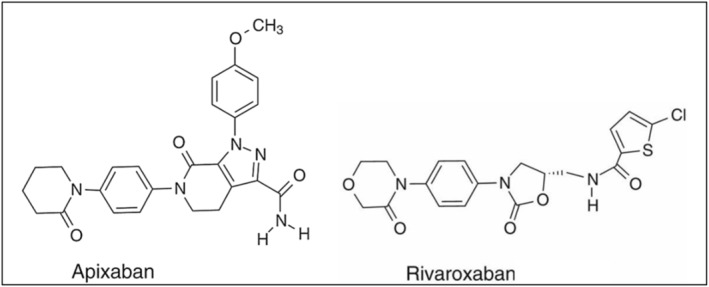
Chemical structure of apixaban (left) and rivaroxaban (right).^28^

While considering cross‐reactivity between rivaroxaban (C_19_H_18_C_l_N_3_O_5_S) and apixaban (C_25_H_25_N_5_O_4_) (Figure 1 **),** it should be noted that both drugs undergo similar metabolic reactions and it is difficult to consider metabolic pathways as responsible for hypersensitivity reactions. Besides metabolism, molecular weight, selectivity/affinity for the receptor, and “L”‐shaped molecule are nearly similar in both agents. Apixaban has a molecular weight of 459.4971 kDa as compared with rivaroxaban (435.881 kDa).^28^, ^29^

Apixaban and rivaroxaban differ in their interactions with factor Xa (FXa) and the prothrombinase complex. Despite sharing an “L”‐shaped structure, they bind differently to the substrate‐binding subsites S1 and S4 within the active site cleft, with varying rigidity. These differences may stem from FXa's lesser dependence on the S1 and S4 binding pockets for apixaban. Additionally, rivaroxaban's greater potency in inhibiting prothrombinase‐induced thrombin generation (2‐fold higher than apixaban) highlights their distinct interactions with the prothrombinase complex. Notably, apixaban and rivaroxaban employ different moieties to bind to FXa: chlorothiophene (rivaroxaban) and p‐methoxybenzene ring (apixaban) occupy the anionic S1 sub‐pocket, while the morpholinone‐bearing nonpolar aryl ring (rivaroxaban) and phenyl‐lactam (apixaban) occupy the aromatic S4 sub‐pocket.^17^, ^30^ Different Structure–Activity Relationship (SAR) and pharmacological activities of the DOACs have been previously described during the COVID‐19 pandemic as it has been suggested that apixaban was 21‐fold more potent than GC376 (control), rivaroxaban, and dabigatran for inhibiting viral M^pro^ activity that may be attributed to more hydrogen bonding interaction with the substrate as well as the ability to bind to an allosteric site on M^pro^ in apixaban, a capability not observed with rivaroxaban and dabigatran.^31^

While hypersensitivity reactions to DOACs are infrequent, they can substantially impact patient quality of life. Diagnostic tools like skin patch tests and specific immunoglobulin E tests can help establish links between DOACs and adverse reactions. These techniques provide essential insights and help identify which DOACs may trigger hypersensitivity, enhancing patient safety and care. Ongoing research into these methods is crucial for better understanding and managing hypersensitivity reactions associated with DOACs. Another point to consider is the approach to switching from one DOAC to another. When switching between DOACs, it's recommended to discontinue the current regimen and start the new one at the next scheduled dose, without overlap.^32^ Given that apixaban is dosed twice daily with a half‐life of about 12 hours, and assuming normal renal function, a once‐daily dose of rivaroxaban can be safely administered 12 hours after the last dose of apixaban.^33^ Conversely, when switching from rivaroxaban to apixaban, the apixaban can be administered 24 hours after the last dose of rivaroxaban.^33^

In this case report, a patient with a maculopapular rash and itching from rivaroxaban was successfully switched to apixaban. The lack of comprehensive data on managing hypersensitivity reactions to DOACs, especially concerning cross‐reactivity, highlights a significant knowledge gap. This successful switch highlights the need for more research into cross‐reactivity and management strategies for hypersensitivity in patients treated with DOACs, potentially leading to safer and more effective treatment options for those experiencing similar adverse reactions.

The precise management of hypersensitivity reactions and the cross‐reactivity among various direct oral anticoagulants remain unclear. In these cases, Strategies for switching between them may be feasible and could be considered, provided there is close monitoring of the patient for any adverse effects based on individual patient responses and tolerability.

## AUTHOR CONTRIBUTIONS

**Keyhan Mohammadi:** Conceptualization; data curation; formal analysis; funding acquisition; investigation; methodology; project administration. **Ramin Ansari:** Conceptualization; data curation; formal analysis; funding acquisition. **Shakila Yaribash:** Investigation; methodology; project administration.

## FUNDING INFORMATION

The authors did not receive funding for this study.

## CONFLICT OF INTEREST STATEMENT

All listed authors were enabled access to relevant data and had a role in writing the manuscript. This project received no specific grant from any funding agency in the public, commercial, or not‐for‐profit sectors.

## CONSENT

Written informed consent was obtained from the patient to publish this report in accordance with the journal's patient consent policy.

## Data Availability

All data generated or analyzed during this study are available as part of the article, and no additional source data are required.
